# Mortality after cancer diagnosis among children with congenital heart disease in Denmark and Sweden

**DOI:** 10.1093/jnci/djaf010

**Published:** 2025-01-17

**Authors:** Christina-Evmorfia Kampitsi, Line Kenborg, Hanna Mogensen, Olof Broberg, Ingrid Glimelius, Friederike Erdmann, Jeanette Falck Winther, Maria Feychting, Giorgio Tettamanti

**Affiliations:** Unit of Epidemiology, Institute of Environmental Medicine, Karolinska Institutet, 171 77 Stockholm, Sweden; Childhood Cancer Research Group, Danish Cancer Institute, 2100 Copenhagen, Denmark; Unit of Epidemiology, Institute of Environmental Medicine, Karolinska Institutet, 171 77 Stockholm, Sweden; Department of Pediatric Cardiology, Skane University Hospital, 221 85 Lund, Sweden; Department of Clinical Sciences, Pediatrics, Lund University, 221 85 Lund, Sweden; Department of Immunology, Genetics and Pathology, Cancer Precision Medicine, Uppsala University, 751 85 Uppsala, Sweden; Research group Aetiology and Inequalities in Childhood Cancer, Division of Childhood Cancer Epidemiology, Institute of Medical Biostatistics, Epidemiology and Informatics (IMBEI), University Medical Center of the Johannes Gutenberg University Mainz, 55131 Mainz, Germany; Department of Prevention and Evaluation, Leibniz Institute for Prevention Research and Epidemiology—BIPS, 28359 Bremen, Germany; Childhood Cancer Research Group, Danish Cancer Institute, 2100 Copenhagen, Denmark; Unit of Epidemiology, Institute of Environmental Medicine, Karolinska Institutet, 171 77 Stockholm, Sweden; Unit of Epidemiology, Institute of Environmental Medicine, Karolinska Institutet, 171 77 Stockholm, Sweden; Department of Molecular Medicine and Surgery, Karolinska Institutet, 171 76 Stockholm, Sweden

## Abstract

**Background:**

Recent decades have witnessed tangible improvements in childhood cancer survival. However, the prognosis for children with congenital heart disease (CHD), the most prevalent birth defect, remains unclear. Due to improved survival of CHD and childhood cancer, evaluating outcomes within this intersection is important for clinical practice. We aimed to assess mortality post-cancer diagnosis among children with CHD.

**Methods:**

We conducted a study on the population of Denmark and Sweden, born 1970-2014, with a cancer diagnosis before age 20 in the national cancer registers (end of follow-up 2015; n = 20 665). CHD diagnoses (n = 397) and recorded deaths were retrieved from national health registers. We evaluated the effect of CHD on 5-year mortality post-cancer diagnosis fitting Cox proportional hazards regression.

**Results:**

When excluding children with Down syndrome, children with CHD had a higher 5-year mortality post-cancer diagnosis compared to children without (hazard ratio [HR] 1.48, 95% confidence interval [CI] = 1.18 to 1.86). This was particularly notable in children with lymphoma (HR = 2.17, 95% CI = 1.11 to 4.25) and neuroblastoma (HR = 2.39, 95% CI = 1.11 to 5.15). In more recent decades (post-1990), children with CHD had similar 5-year mortality as their counterparts without, except for children diagnosed with lymphoma, where mortality remained elevated (HR = 3.37, 95% CI = 1.65 to 6.89).

**Conclusions:**

In this large, register-based cohort study, children with CHD fared worse post-cancer diagnosis—particularly lymphoma and neuroblastoma. While a more positive trend emerged in recent years, lymphoma-related mortality remained disproportionately high among children with CHD, underscoring the need for continued research and interventions to improve outcomes for this vulnerable group.

## Introduction 

Over the past decades, childhood cancer survival has improved substantially in high income countries, owing to access to improved diagnostics and effective multidisciplinary treatment and supportive care. As a result, 5-year overall survival has increased to over 80% in Europe, albeit with persisting disparities between cancer types and geographical regions.^1^ It is unknown, however, to what extent this marked improvement extends to children with birth defects—one of the conditions most consistently associated with an increased childhood cancer risk.^2^

Congenital heart disease (CHD), the most common birth defect, affects approximately 1% of all live births, while its prevalence has been on the rise.^3^ This increased prevalence is attributed to diagnostic and therapeutic breakthroughs that have largely transformed even severe CHD from a nearly universally fatal ailment into a manageable chronic condition.^4^ Nowadays, about 97% of children with CHD reach adulthood.^5^ However, the distribution of morbidity and mortality in this patient group is not adequately understood, especially concerning their non-cardiac complications. A growing body of evidence is pointing towards an increased risk of cancer in children with CHD,^2^^,^^6^^,^^7^ yet little is known regarding subsequent survival—despite cancer being a leading cause of death in adults with CHD.^8^

In the wake of improved survival for both CHD and childhood cancer, it becomes increasingly important to assess the post-cancer diagnosis outcomes for these young individuals, to inform decision-making and refine clinical practice. Therefore, we sought to assess the relationship between CHD and mortality post-cancer diagnosis in a large, nationwide cohort of Danish and Swedish children and adolescents, utilizing routinely collected data from national administrative and health registers.

## Methods

### Study design, population, and data sources

The Nordic countries have well-established, high-quality national population and health registries with comprehensive coverage.^9^ A distinguishing feature of these registers is the unique personal identification numbers assigned to all residents and used consistently across registries, enabling precise linkage of information across registries on an individual level. This infrastructure ensures that virtually all individuals can be followed from birth to death, while emigration is limited. Furthermore, all Nordic countries operate under welfare state models characterized by universal, tax-funded healthcare systems. This uniformity in healthcare systems and registers along with sociocultural and socioeconomic similarities across the Nordic countries facilitates the amalgamation of multi-country data into a unified study cohort.

Utilizing this unparalleled data resource, we conducted a cohort study encompassing the child and adolescent populations of Denmark and Sweden, born between 1970 and 2014, who were diagnosed with cancer before the age of 20 years. The cancer diagnoses were retrieved from 1977 until 2014 for Denmark and 1970 to 2015 for Sweden and identified through the Danish and Swedish nationwide cancer registers, which have been operational since 1943 and 1958, respectively.^10^ Reporting of cancer diagnoses is mandatory in both countries. As such, the Danish and Swedish cancer registers are renowned for their high quality and completeness, with minimal instances of underreporting noted.^11^^,^^12^ Leveraging the information obtained from the national cancer registers, we categorized childhood cancer diagnoses according to the International Classification of Childhood Cancer, third edition (ICCC-3), which classifies tumors coded according to the World Health Organization International Classification of Diseases for Oncology third version (ICD-O-3) nomenclature into 12 major diagnostic groups.^13^ Our study encompassed all childhood cancers, with separate consideration of the prevailing types: leukemias (ICCC-3 group I) and separately acute lymphoblastic leukemia (ALL), central nervous system (CNS) tumors (ICCC-3 group III), and lymphomas (ICCC-3 group II). Additionally, given prior research highlighting an increased risk of neuroblastoma in children with CHD,^2^ we extended our investigation to encompass survival after neuroblastoma diagnoses (ICCC-3 group IV). Information on deaths among the study participants was retrieved from the cause of death registers.^14^^,^^15^

Diagnoses of CHD among Danish-born children were retrieved from the Danish national patient register, established in 1977 and including diagnoses from hospitalization and contacts with specialist healthcare.^16^ For Sweden, CHD diagnoses from the Swedish national patient register (initiated in 1964)^17^ were supplemented with data from the national Medical Birth Register (MBR). The Swedish MBR was established in 1973 and records comprehensive information on deliveries, maternal and fetal characteristics, and health conditions detected during perinatal, delivery, or neonatal care.^18^ In both countries, only CHD diagnoses preceding the cancer diagnosis were considered. Therefore, in Denmark, we only considered cancer diagnosed from 1977, which is the earliest time point we can obtain information on CHD diagnoses.

We followed our study population from the first cancer diagnosis until the earliest of the following events: completion of 5 years post-cancer diagnosis, death, emigration, or end of follow-up (December 2015). Information regarding emigrations was sourced from population registers,^19^^,^^20^ while date of death was extracted from the cause of death registers.^14^^,^^15^

The study has been approved by Statistics Denmark and the Regional Ethical Review Board in Stockholm. Informed consent was waived as the analyzed data were de-identified. Research was conducted in compliance with the national requirements of the General Data Protection Regulation (GDPR).

### Statistical analysis

Descriptive statistics were employed to summarize the baseline characteristics of the study cohort. Using Cox proportional hazards regression models with time since cancer diagnosis as the underlying time scale, we estimated hazard ratios (HR) and corresponding 95% confidence intervals (CI) for 5-year mortality after a childhood cancer diagnosis, comparing children with CHD to those without. We considered overall childhood cancer mortality, along with distinct assessment for childhood leukemias (and separately ALL), CNS tumors, lymphomas, and neuroblastoma.

To capture recent trends in survival among children with CHD following a cancer diagnosis, the analysis was reiterated for those diagnosed with cancer after 1990. The analyses were performed both for combined data across countries and individually for each country. We adjusted for assigned sex at birth (male, female), age at cancer diagnosis (<1, 1-4, 5-9, 10-14, and 15-19 years of age), year of cancer diagnosis (1970-1979, 1980-1989, 1990-1999, 2000-2009, and 2010-2014), and maternal age at child’s cancer diagnosis (<30, 30-39, and ≥40 years of age). For the combined analysis, we further adjusted for country at cancer diagnosis. Analyses from 1990 onward were further adjusted for maternal education (lower secondary or less, upper secondary, postsecondary), as consistent data on this variable became available from that time. As Down syndrome is the most prevalent chromosomal birth defect, markedly influences the association between CHD and childhood leukemia risk,^7^ and is strongly linked to both CHD and survival outcomes in childhood leukemia,^21-23^ children with Down syndrome were excluded from the above Cox regression models. Analyses were not performed when fewer than 5 cancer deaths were recorded during follow-up for a specific exposure category. To assess the proportional hazards assumption, we used the Schoenfeld residuals test.

Subsequently, we visually illustrated 5-year survival following a childhood cancer diagnosis plotting Kaplan–Meier survival curves. We initially compared all children with CHD to those without. Then, we refined the comparison by excluding children with Down syndrome from both groups. Ultimately, we extended the comparison cohorts to encompass children with varying combinations of CHD and Down syndrome. In additional analyses we fitted Cox models to assess the potential interaction between CHD and Down syndrome in relation to mortality after childhood leukemia and childhood ALL. This is particularly pertinent as Down syndrome has been demonstrated as a robust risk factor specifically for heightened childhood ALL mortality.^22^^,^^23^ Although other congenital anomalies are not strongly associated with both CHD and childhood cancer survival, neurofibromatosis—a neurocutaneous syndrome—has been linked to certain congenital anomalies, including CHD, as well as improved survival in optic pathway gliomas.^24^^,^^25^ Therefore, we conducted a sensitivity analysis excluding individuals with the 2 most common neurocutaneous syndromes: neurofibromatosis and tuberous sclerosis complex.

In all analyses, 95% confidence intervals were 2-sided. Data were prepared with SAS 9.4, and all analyses were conducted with STATA 14.2.

## Results

A total of 20 665 children and adolescents with cancer were included in this study. Among them, 7649 were residing in Denmark and 13 016 in Sweden. In the entire cohort, 397 individuals had received a CHD diagnosis, with 118 diagnoses stemming from Denmark and 279 from Sweden (Table 1). More than 20% of children with CHD also had a Down syndrome diagnosis. Moreover, a higher proportion of children with CHD received their cancer diagnosis within the first 4 years of life, whereas children without CHD tended to be older at cancer diagnosis.

**Table 1.** Baseline characteristics of children born 1970-2014 and diagnosed with cancer in Denmark and Sweden by CHD status.^a^

	CHD-free, n (%)	CHD, n (%)
Sex		
Male	11 001 (54.3)	191 (48.1)
Female	9267 (45.7)	206 (51.9)
Maternal age, years		
<30	3637 (18.2)	69 (17.4)
30-49	8619 (43.0)	172 (43.4)
≥40	7776 (38.8)	155 (39.2)
Age at cancer diagnosis, years		
<1	1607 (7.9)	59 (14.9)
1-4	5752 (28.4)	137 (34.5)
5-9	3920 (19.3)	69 (17.4)
10-14	3713 (18.3)	47 (11.8)
15-19	5276 (26.1)	85 (21.4)
Period of cancer diagnosis		
1970-1979	1269 (6.3)	12 (3.0)
1980-1989	3846 (19.0)	48 (12.1)
1990-1999	5795 (28.6)	99 (25.0)
2000-2009	5760 (28.4)	128 (32.2)
2010-2015	3598 (17.7)	110 (27.7)
Down syndrome	101 (0.5)	91 (22.9)
Country		
Denmark	7531 (37.2)	118 (29.7)
Sweden	12 737 (62.8)	279 (70.3)

a Cancer diagnoses were retrieved 1977-2014 for Denmark and 1970-2015 for Sweden.

Abbreviation: CHD = congenital heart disease.

**Figure 1.**
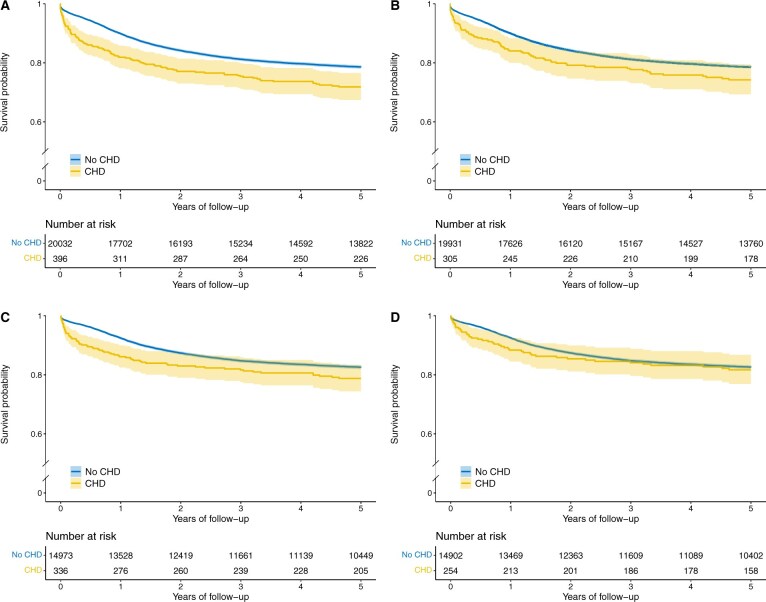
Five-year overall survival from childhood cancer. Row 1 (A and B) displays survival for all cancer diagnoses across the entire study period, while Row 2 (C and D) shows survival for cancers diagnosed after 1990. (A) and (C) compare survival between children with CHD and those without CHD. (B) and (D) compare survival between children with CHD, excluding those with DS, and children without CHD or DS. Abbreviations: CHD = congenital heart disease; DS = Down syndrome.

**Figure 2.**
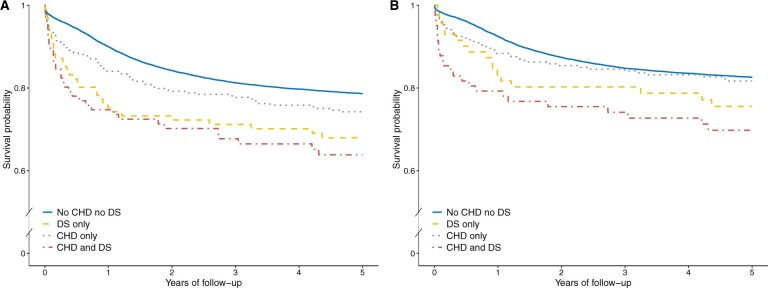
Five-year overall survival from childhood cancer among children with varying combinations of CHD and DS. (A) shows survival for all cancer diagnoses across the entire study period, while (B) focuses on cancers diagnosed after 1990. The risk table is omitted due to Statistics Denmark regulations requiring a minimum difference of 5 individuals between each year to prevent identification of individuals. Abbreviations: CHD = congenital heart disease; DS = Down syndrome.

Survival following any childhood cancer diagnosis is illustrated in Figures 1 and 2. We observed consistently poorer survival post-cancer diagnosis among children with CHD (Figure 1, A). This disparity in survival between children with and without CHD became less pronounced when children with Down syndrome were excluded (Figure 1, B). Notably, the poorest survival was observed among children with both CHD and Down syndrome (Figure 2, A). This finding was largely driven by leukemia, specifically ALL. The HR of mortality after an ALL diagnosis among children with both CHD and Down syndrome was 3.34 (95% CI = 1.95 to 5.72) (Supplementary Table 1).

After excluding children with Down syndrome, the overall HR for 5-year mortality after any cancer diagnosis among children with CHD was 1.48 (95% CI = 1.18 to 1.86), compared to children without CHD (Table 2). Five-year mortality among children with CHD was most pronounced in neuroblastoma (HR = 2.39; 95% CI = 1.11 to 5.15) and lymphoma (HR = 2.17; 95% CI = 1.11 to 4.25). While 5-year leukemia mortality was also increased (HR = 1.52; 95% CI = 1.04 to 2.23), this finding was driven solely by Danish data (HR = 2.69; 95% CI = 1.60 to 4.52). Excluding individuals with neurofibromatosis or tuberous sclerosis complex did not materially impact the results for the combined data (Supplementary Table 2). Cancer was the leading cause of death among those who died during the study period, both among children with (81.1%) and without (91.1%) CHD.

**Table 2.** Five-year mortality after cancer diagnosis among children born 1970 to 2014 with CHD in Denmark and Sweden (HRs and 95% CIs).

	Combined data		Denmark	Sweden
	CHD/no CHD, No. of deaths	HR (95% CI)	HR (95% CI)	HR (95% CI)
All cancers	75/4136	1.48 (1.18 to 1.86)	1.53 (1.04 to 2.25)	1.44 (1.08 to 1.91)^a^
Leukemia	27/1212	1.52 (1.04 to 2.23)	2.69 (1.60 to 4.52)	0.96 (0.54 to 1.70)
ALL	12/710	1.29 (0.73 to 2.28)	2.32 (1.03 to 5.25)	0.85 (0.38 to 1.89)
CNS	17/1226	1.12 (0.70 to 1.82)	0.91 (0.43 to 1.92)^a^	1.37 (0.73 to 2.55)
Lymphoma	9/314	2.17 (1.11 to 4.25)	—^b^	—^b^
Neuroblastoma	7/298	2.39 (1.11 to 5.15)	—^b^	—^b^

Children with Down syndrome are excluded from the analyses. Adjusted for sex, age at cancer diagnosis, year of cancer diagnosis, and maternal age. Combined data are further adjusted for country of diagnosis.

Abbreviations: ALL = acute lymphoblastic leukemia; CHD = congenital heart disease; CI = confidence interval; CNS = central nervous system; HR = hazard ratio.

a Proportional hazards assumption not met.

b When fewer than 5 cancer deaths were recorded during follow-up, analyses were not performed.

When restricting the analysis to children diagnosed with cancer from 1990 onwards, substantial improvements in survival were evident among children with CHD (Figure 1, C). After exclusion of children with Down syndrome, cancer survival among children with CHD aligned with that of children without CHD after the 2-year threshold (Figure 1, D). Children affected by both CHD and Down syndrome continued to exhibit the poorest survival, following both cancer overall and leukemia; yet their survival was improved compared to the entirety of the study period (Figure 2, B and Supplementary Table 3). Survival following a lymphoma diagnosis persisted as comparatively poorer among children with CHD (Table 3).

**Table 3.** Five-year mortality after cancer diagnosed from 1990 onwards among children with CHD in Denmark and Sweden (HRs and 95% CIs).

	Combined data		Denmark	Sweden
	CHD/no CHD, No. of deaths	HR (95% CI)	HR (95% CI)	HR (95% CI)
All cancers	41/2313	1.16 (0.85 to 1.57)	1.08 (0.62 to 1.86)	1.20 (0.82 to 1.73)^a^
Leukemia	10/591	0.98 (0.52 to 1.82)	1.79 (0.73 to 4.38)	0.67 (0.28 to 1.63)
ALL	5/332	0.93 (0.39 to 2.26)	—^b^	—^b^
CNS	10/704	0.98 (0.53 to 1.84)	—^b^	—^b^
Lymphoma	8/173	3.37 (1.65 to 6.89)	—^b^	—^b^

Children with Down syndrome are excluded from the analyses. Adjusted for sex, age at cancer diagnosis, year of cancer diagnosis, maternal age, and maternal education. Combined data are further adjusted for country of diagnosis.

Abbreviations: ALL = acute lymphoblastic leukemia; CHD = congenital heart disease; CI = confidence interval; CNS = central nervous system; HR = hazard ratio.

a Proportional hazards assumption not met.

b When fewer than 5 cancer deaths were recorded during follow-up, analyses were not performed.

## Discussion

In this population-based cohort study, encompassing almost 21 000 children diagnosed with cancer in Denmark and Sweden, we observed an elevated 5-year mortality following a cancer diagnosis among children with CHD compared to children without. Notably, lymphoma and neuroblastoma emerged as the primary contributors to this association. Furthermore, when including children with Down syndrome alongside those with CHD, we observed that children with both Down syndrome and CHD exhibited the poorest survival post-cancer diagnosis, primarily driven by death following an ALL diagnosis. While the disparity in post-cancer survival between children with and without CHD has shown a notable reduction in recent decades (post-1990), mortality following a lymphoma diagnosis persisted as comparatively elevated among children with CHD.

To our knowledge, no prior studies have specifically examined post-cancer diagnosis mortality in children with CHD. However, noteworthy findings from related research align with our finding of increased mortality post-cancer diagnosis in children with CHD. A smaller register-based study conducted in Oklahoma identified an elevated cancer-related mortality within the broader context of birth defects.^26^ Moreover, Down syndrome has consistently shown associations with elevated mortality in ALL, coupled with decreased mortality from other leukemias.^27^^,^^28^ In our study, we observed the most pronounced decline in survival among children with CHD during the first year after the cancer diagnosis. This observation is also corroborated from studies investigating survival post-leukemia diagnosis in children with Down syndrome.^28^ Moreover, our finding of a notably high post-lymphoma diagnosis mortality among children with CHD is in line with previous results from a retrospective multinational study on event-free survival following non-Hodgkin lymphoma diagnosis in children with pre-existing conditions. This investigation, conducted by the European Intergroup for Childhood non-Hodgkin Lymphoma and the international Berlin-Frankfurt-Münster Study Group, revealed a marked proportion of therapy-related toxicities and subsequent deaths in children with pre-existing conditions.^29^ Yet, it is worth noting that these pre-existing conditions encompassed a diverse range of congenital malformations, neurocutaneous syndromes, and other cancer predisposition syndromes, making it challenging to disentangle the specific effect of each condition. Finally, a recent Swedish register-based matched cohort study reported that post-cancer mortality among CHD patients did not differ significantly from that of non-CHD patients when individuals with genetic syndromes were excluded.^30^ However, this study combined children and young adults and did not separately analyze specific cancer diagnoses, despite the considerable variation in treatment protocols and outcomes across different cancer types.

It becomes apparent, therefore, that beyond the well-studied relationship between Down syndrome and post-childhood leukemia mortality, studies assessing the impact of other birth defects on survival after childhood cancer are lacking. Nonetheless, our finding of improved survival outcomes for children with CHD and leukemia post-1990 likely reflects advancements in pediatric cancer treatment, including more precise risk stratification and personalized therapies.^31^ In particular, reductions in the use and intensity of radiotherapy have likely helped mitigate cardiotoxicity—a crucial concern for CHD patients.^32^ Furthermore, cardiovascular comorbidities have been associated with a higher risk of lymphoma-specific mortality in prior studies;^33^ in the present study, several mechanisms likely underpin the persistent association between CHD and poorer survival post-childhood cancer diagnosis. The complexity of certain congenital heart defects might influence decision-making regarding highly effective yet cardiotoxic therapies.^34^ Doxorubicin, one of the most effective chemotherapeutic agents in the first-line treatment for lymphoma,^35^ is known for its cardiotoxic side effect profile;^36^ as a result, children with CHD may receive reduced doses or alternative therapies that, while safer, may also be less effective. Similarly, later-stage treatments, such as either autologous or allogeneic stem cell transplants, may be avoided due to concerns over the general frailty and comorbidities in children with CHD. Moreover, concurrent heart failure, pulmonary hypertension, and arrythmias might render CHD patients more sensitive to complications such as sepsis or acute cardiotoxicity during cancer therapies.^37^^,^^38^ Such cardiotoxicity may necessitate further dose reductions, treatment delays, or even the omission of chemotherapy cycles, increasing the risk of cancer progression or relapse. Also, post-treatment, an already frail circulatory status could be more susceptible to different late toxic effects from chemo- and radiotherapy causing death. Other conditions prevalent in the CHD population, such as 22q11.2 deletion syndrome (DiGeorge syndrome), might also contribute to such risks.^39^ Given the increased susceptibility of children with CHD to cancer development, particularly lymphoma,^7^ achieving a more comprehensive understanding of cancer outcomes among these children has the potential to greatly enhance their clinical management, including the development of specific treatment protocols.

Leveraging the well-established health and population registers of Denmark and Sweden, we achieved population-wide coverage and access to prospectively collected, objective, and standardized data on cancer and CHD diagnoses for children born during a period spanning over 4 decades, minimizing the potential for selection and information bias. This extensive follow-up period enabled us to capture temporal trends in survival. Moreover, the register-based design enabled us to consider several relevant covariates, including the identification of children with Down syndrome.

While our study offers valuable insights, it is not without limitations. Although we delve into the impact of CHD on post-childhood cancer diagnosis mortality, it is important to acknowledge the inability to isolate the effect of childhood cancer itself on the mortality of children with CHD. Consequently, a portion of the heightened mortality risk could potentially be attributed to the underlying congenital heart malformation. Despite considerable improvements in CHD survival, the risk of mortality among children with CHD remains higher compared to the general population.^5^ Nevertheless, it is important to note that the increased mortality estimates were consistently noted only post-lymphoma diagnosis, rather than across all cancer groups, and there is no compelling reason to posit that the underlying heart malformation would disproportionally impact these specific cases. Additionally, our access to CHD diagnoses in Danish data was confined to the national patient register. Consequently, there is a possibility that we may have missed CHD diagnoses made at birth if they were not reiterated later in life. In the case of Swedish data, a fraction of the total CHD diagnoses, slightly less than one-fifth, was sourced solely from the MBR. This potential underdiagnosis in the Danish data may have diluted our observed effect estimates. It bears mentioning that a lower percentage of CHD diagnoses in Denmark could also be attributed to few Down syndrome live births, following early adoption of widely available prenatal screening for Down syndrome.^40^ Moreover, our study was unable to explore post-cancer mortality in specific treatments due to lack of detailed clinical information on chemotherapy regimens and associated toxicities. Furthermore, while the present study encompassed all cancer cases in children and adolescents born between 1970 and 2014 across 2 countries (diagnosed 1977-2014 in Denmark and 1970-2015 in Sweden), the rarity of CHD and childhood cancer, coupled with overall high survival rates after childhood cancer resulted in limited numbers for stratified analyses by ICCC group and reduced precision in some subgroup effect estimates. The limited number of deaths also precluded examination of outcomes by CHD complexity, which would have been particularly insightful given our previous findings of increased lymphoma risk among children with complex CHD.^7^ Another limitation is that, while the proportional hazards assumption was satisfied for the main exposure (CHD) in analyses combining data from both countries, occasional violations were observed in single-country analyses. It is important to note that, given the extended time span of our study, Hazard Ratios should be interpreted as the average Hazard Ratio across the entire study period. Finally, it is worth noting that Denmark and Sweden are countries benefitting from universal healthcare that is free of charge for children. Therefore, our results may not be directly generalizable to healthcare settings with differing access to care, less centralization, or varying rates of CHD survival.

In conclusion, this extensive population-based 2-country wide cohort study highlights an increased 5-year mortality risk among children with CHD following a cancer diagnosis. Lymphoma and neuroblastoma emerge as notable drivers of this heightened risk. While advancements in diagnostics and treatment seem to have narrowed the gap in survival between children with and without CHD in more recent decades, the persistence of elevated mortality following a lymphoma diagnosis underscores the need for continued research and interventions to improve outcomes for this vulnerable group. This may include the introduction of new, effective treatment regimens with improved toxicity profiles. Future research should investigate whether the increased post-lymphoma mortality is impacted by CHD complexity, age at cancer diagnosis, or specific treatment regimens.

## Supplementary Material

djaf010_Supplementary_Data

## Data Availability

The data analyzed in this study are remotely stored in a secure platform at Statistics Denmark. Pseudonymized personal data were acquired from national registry authorities following ethical approval (where applicable) and secrecy assessment. Danish and Swedish laws and regulations do not allow sharing of personal sensitive data, which can only be made available for researchers who fulfill the legal requirements for access to such data. Please contact Line Kenborg (kenborg@cancer.dk) with inquiries regarding data access.
