# Supplementary material for: Mortality after cancer diagnosis among children with congenital heart disease in Denmark and Sweden
Source: J Natl Cancer Inst. 2025 Jan 17;117(6):1134–41. doi: 10.1093/jnci/djaf010 (PMC12145920; doi:10.1093/jnci/djaf010)
Supplement: djaf010_Supplementary_Data [file djaf010_supplementary_data.pdf]

SUPPLEMENTARY MATERIAL

Supplementary Table 1. Five-year mortality after leukemia diagnosis among children born 1970 to 2014 with CHD in Denmark and Sweden (HRs and 95% CIs)

	Combined data		Denmark	Sweden
	Deaths, n	HR (95% CI)	HR (95% CI)	HR (95% CI)
		**Leukemia**		
CHD-DS				
No CHD or DS	1,212	1 [Reference]	1 [Reference]	1 [Reference]
DS	29	1.30 (0.89–1.89)	1.62 (0.67–3.95)	1.28 (0.84–1.94)
CHD	27	1.53 (1.04–2.24)	2.67 (1.59–4.49)	0.96 (0.54–1.71)
CHD and DS	29	2.45 (1.69–3.57)	3.53 (1.80–6.91)	2.26 (1.44–3.55)
		**ALL**		
CHD-DS				
No CHD or DS	710	1 [Reference]	—*	1 [Reference]
DS	12	1.30 (0.73–2.32)	—*	1.18 (0.60–2.32)
CHD	12	1.29 (0.73–2.28)	—*	0.84 (0.38–1.89)
CHD and DS	14	3.34 (1.95–5.72)	—*	2.53 (1.24–5.16)

Adjusted for sex, age at cancer diagnosis, year of cancer diagnosis, and maternal age.
Combined data is further adjusted for country of diagnosis.

*When fewer than 5 cancer deaths were recorded during follow-up, analyses were not performed.

Abbreviations:
CHD, congenital heart disease ; HR, hazard ratio ; CI, confidence interval ; ALL, acute lymphoblastic leukemia

Supplementary Table 2. Five-year mortality after cancer diagnosis among children born 1970 to 2014 with CHD in Denmark and Sweden (HRs and 95% CIs)—additional exclusions

	Combined data	
	CHD/no CHD no. of deaths	HR (95% CI)
All cancers	73/4,095	1.50 (1.19–1.89)
Leukemia	27/1,210	1.52 (1.04–2.23)
ALL	12/710	1.29 (0.73–2.28)
CNS	15/1,211	1.20 (0.72–2.00)
Lymphoma	9/313	2.18 (1.12–4.27)
Neuroblastoma	7/298	2.41 (1.12–5.19)

Children with Down syndrome, neurofibromatosis and tuberous sclerosis are excluded from the analyses.

Adjusted for sex, age at cancer diagnosis, year of cancer diagnosis, maternal age, and country of diagnosis.

Abbreviations:
CHD, congenital heart disease ; HR, hazard ratio ; CI, confidence interval ; CNS, central nervous system ; ALL, acute lymphoblastic leukemia

Supplementary Table 3. Five-year mortality after leukemia diagnosed from 1990 onwards among children with CHD in Denmark and Sweden (HRs and 95% CIs)

	Combined data	
	Deaths, n	HR (95% CI)
**Leukemia**		
CHD-DS		
No CHD or DS	591	1 [Reference]
DS	12	1.44 (0.81–2.57)
CHD	10	1.00 (0.53–1.87)
CHD and DS	20	1.93 (1.23–3.04)
**ALL**		
CHD-DS		
No CHD or DS	332	1 [Reference]
DS	8	2.78 (1.36–5.65)
CHD	5	0.95 (0.39–2.31)
CHD and DS	12	3.15(1.74–5.69)

Adjusted for sex, age at cancer diagnosis, year of cancer diagnosis, maternal age, maternal education, and country of diagnosis.

Analyses for individual country data were not performed, as fewer than 5 cancer deaths were recorded during follow-up.

Abbreviations:
CHD, congenital heart disease ; HR, hazard ratio ; CI, confidence interval ; ALL, acute lymphoblastic leukemia
